# The effect of patent *Dictyocaulus viviparus* (re)infections on individual milk yield and milk quality in pastured dairy cows and correlation with clinical signs

**DOI:** 10.1186/s13071-017-2602-x

**Published:** 2018-01-08

**Authors:** Katharina May, Kerstin Brügemann, Sven König, Christina Strube

**Affiliations:** 1 Institute for Parasitology, Centre for Infection Medicine, University of Veterinary Medicine Hannover, Buenteweg 17, 30559 Hannover, Germany; 2 Institute of Animal Breeding and Genetics, Justus-Liebig-University of Gießen, 35390 Gießen, Germany

**Keywords:** *Dictyocaulus viviparus*, Bovine lungworm, Larval shedding, Milk production, Milk quality, Dairy cows, Bulk tank milk, ELISA

## Abstract

**Background:**

Infections with the bovine lungworm *Dictyocaulus viviparus* might lead to reduced milk production and detrimental impacts on milk quality resulting in considerable economic losses in dairy farming.

**Methods:**

In the presented field study, 1988 faecal samples were collected from 1166 Black and White dairy cows allocated in 17 small and medium-sized German grassland farms. Faecal samples were collected in summer and autumn 2015 to assess *D. viviparus* larvae excretion. Test-day records were used to estimate the association between patent *D. viviparus* infections in individual cows and the milk production parameters milk yield, milk protein and milk fat content by using linear mixed models. Bulk tank milk (BTM) samples from each farm and individual milk samples from those cows which were excreting larvae in summer were collected in autumn. In addition, occurrence of the clinical symptom “coughing” was noted in individual cows during autumn sampling to determine its association with patent lungworm infections.

**Results:**

Patent *D. viviparus* infections were found on 23.5% (4/17) of farms with a prevalence at the individual cow level of 0.9% (9/960) in summer and 3.4% (35/1028) in autumn. No BTM sample exceeded the BTM ELISA cut-off value of 0.410 optical density ratio (ODR), the mean value was 0.168 ODR. Only one individual milk sample exceeded the individual milk ELISA cut-off value of 0.573 ODR (mean value of 0.302 ODR). A patent *D. viviparus* infection status was associated with a lower average daily milk yield of 1.62 kg/cow/day (*P* = 0.0406). No significant association was found with milk protein or fat content representing milk quality parameters. Coughing was observed in 5.9% (61/1028) of cows. Of the coughing cows, only 4.9% (3/61) had a patent lungworm infection. Fisher’s exact test showed no significant difference between infected and non-infected coughing cows.

**Conclusions:**

Farmers and veterinarians should be aware that patent lungworm (re)infections in dairy cows reduce milk yield, despite the absence of clinical signs. Furthermore, if dairy cows present with coughing, other differential diagnoses need to be considered in addition to dictyocaulosis.

**Electronic supplementary material:**

The online version of this article (10.1186/s13071-017-2602-x) contains supplementary material, which is available to authorized users.

## Background

The bovine lungworm *Dictyocaulus viviparus* is the causative agent of parasitic bronchitis in first season grazing calves but also in adult dairy cows [1–3]. The infection can lead to subclinical or severe clinical disease with symptoms like coughing, dyspnea or even death [4, 5]. Generally, acquired immunity lasts for 6–12 months in the absence of reinfections [6]. However, in immune cattle, high infection doses and consequently a large number of larvae invading the lungs can result in the reinfection syndrome: a severe immune-mediated inflammatory response [5, 7, 8]. High infection rates up to 80% were observed in dairy herds in the Netherlands based on faecal examinations as well as milk and serum antibody levels [9, 10]. In Flanders, Belgium, 19.6% of dairy herds were tested positive using bulk tank milk (BTM) samples [11]. Similarly, in the northern German region East Frisia, BTM ELISA showed 21.1% of dairy herds to be seropositive for *D. viviparus* on at least one sampling occasion in 2008 [12]. For the whole of Germany, Schunn et al. [2] reported a BTM seroprevalence of 17.1%, with rates up to 31.2% in the federal state Saxony-Anhalt. In Ireland, a BTM seroprevalence of 62.8% was noted [13].

Holzhauer et al. [5] reported considerable economic losses in two lungworm-infected Dutch dairy herds, such as a decrease in milk production, with estimated costs between 160 and 170 € per cow during a lungworm outbreak. The first herd-level evaluation of the relationship between *D. viviparus* infections and milk production parameters (milk yield, milk protein content, milk fat content) assessed by BTM ELISA was conducted by Dank et al. [14]. In this study, *D. viviparus* positive herds showed a decline in milk production between 1.01 and 1.68 kg/cow/day, and lower milk fat content in comparison to herds with a negative BTM ELISA result. Recently, another study reported a significant decline of 0.50 kg/cow/day in annual average milk yield and a decline of 0.02% for annual average milk protein and fat content, when connecting these parameters with BTM ELISA results [15]. However, to our knowledge there is a substantial gap addressing the association between *D. viviparus* infection status and milk production parameters on individual dairy cow level. Furthermore, faecal examination provides a better indicator for a current lungworm infection than *D. viviparus* antibody titres, which persist up to 7 months [16, 17], whereas lungworm infections are usually eliminated 60 to 90 days post-infection [18]. Another problem associated with serological assays was pointed out by Strube et al. [19], who showed that the magnitude and duration of antibody titres following reinfections were reduced as compared to primary infections, or even entirely absent. Hence, it may be difficult to detect *D. viviparus* reinfections by using ELISA in individual cows or BTM samples [5, 19]. Therefore, presence of clinical symptoms such as coughing is often used as a gold standard for lungworm infection in epidemiological studies [15, 20]. Charlier et al. [15] observed an increase in the optical density ratio (ODR) of 0.04 in those herds where coughing was detected. However, this increase is rather low and coughing may also be due to other infective or non-infective agents. Such other agents are of particular importance in serological studies, as cattle may have already recovered from dictyocaulosis while antibodies still persist. To date, no study has assessed the correlation of coughing with patent lungworm infections on individual cow level. Thus, the main objective of the presented study was to estimate the association between patent *D. viviparus* infections and milk production parameters such as milk yield, milk protein and milk fat content in individual dairy cows. Another aim was to assess the relationship between individual faecal examinations with (i) coughing as clinical symptom, and (ii) *D. viviparus* BTM antibody levels.

## Methods

### Farms and animals

The present study was conducted with 17 German dairy farms (13 organic, four conventional) with a strong focus on dairy cow grazing (> 8 h per day). Farms were located in four German federal states, two farms in Hesse, eight farms in Lower Saxony, five farms in North Rhine-Westphalia and two farms in Schleswig-Holstein. The herd size ranged from 19 to 215 dairy cows (mean: 72 cows) per farm. Requirement for selection was that herds were not treated with anthelmintics in the sampling year and access to pasture started not later than 1st of June. Four different genetic lines of Black and White cattle were included in the study following a cross-classified design (several lines per herd): HF-NZ (72 cows) = German Holstein cow (GHC) × New Zealand Holstein sires; HF-GHm (639 cows) = GHC × German Holstein sires with high breeding values for milk yield; HF-GHp (70 cows) = GHC × German Holstein sires selected for pasture conditions and DSN (363 cows) = local Black and White dual-purpose cows of the Deutsche Schwarzbunte Niederungsrind, the founder of the current Holstein breed. Furthermore, the study comprised some Crosses (22 cows) = GHC × Jersey, Angler or beef cattle sires.

### Faecal sampling and examination

In July (summer) and September (autumn) 2015, 1988 faecal samples of 1166 cows were collected rectally for examination of lungworm larvae. Collected samples were instantly cooled to 4 °C and transported within 3 h of collection to the Institute for Parasitology, University of Veterinary Medicine Hannover. In summer, 16 farms were visited and 17 farms in autumn. The interval between both faecal samplings was approximately 8 weeks per farm. In total, 960 faecal samples in summer and 1028 in autumn were processed with the Baermann technique [21] using 40 g faeces per cow. Funnels were lettered with ear tag number of the single cows and larvae were allowed to migrate for approximately 18 h before microscopic examination. Repeated measurements were feasible for 820 cows. Cows with only one parasitological examination were dry cows on inaccessible pastures or cows which were sold or died.

### Herd management and occurrence of coughing

All farmers answered to a written questionnaire at the first visit to obtain information about herd management factors (e.g. herd size, purchase of new cows, anthelmintic treatment in the past or treatment of young stock, diagnostic tools to detect lungworm infections in the herd). In autumn, it was additionally noted if farmers observed coughing as a possible clinical symptom of dictyocaulosis in the herd or in individual cows. Additionally, the presence of coughing (CS+, positive coughing status; CS-, negative coughing status) in individual cows was recorded by two investigators for approximately 2–3 h during the autumn farm visit. If coughing was observed in at least one individual in the herd, the herd was classified to a CS+ status.

### MSP-ELISA

BTM samples were taken in autumn (September) and transported at a steady temperature of 4 °C to the Institute for Parasitology, University of Veterinary Medicine Hannover, Germany. After arriving, BTM samples were centrifuged at 2000× *g* for 15 min. Afterwards, the fat layer was removed and milk was stored at -20 °C until tested with the BTM ELISA based on recombinant major sperm protein (MSP) as antigen [8]. In addition, individual milk samples were collected in autumn from those cows which had excreted larvae in summer. *Dictyocaulus viviparus* antibody levels were analysed using the individual milk ELISA as described by Fiedor et al. [17]. BTM samples were assessed with the cut-off value 0.410 ODR, individual milk samples with the cut-off value 0.573 ODR [8, 17].

### Milk production data

Test-day and individual dairy cow data, such as parity, days in milk (DIM) or genetic line were provided from the National Genetic Evaluation Center (Vereinigte Informationssysteme Tierhaltung, VIT, Verden, Germany). Analysed individual test-day dairy cow production data were first test-day records after parasitological examination for milk yield (kg milk/cow/day), milk protein content (%) and milk fat content (%).

### Statistical analysis

The statistical software SAS version 9.4 [22] was used to perform statistical analyses. Descriptive statistics were analysed with FREQ and MEANS procedures. The cows were classified by their *D. viviparus* infection status as assessed with the Baermann technique (faecal larvae count [FLC] ≥ 1 = positive and FLC = 0 = negative). For estimations concerning coughing, our own coughing observations (CS+ or CS-) as opposed to those of the farmer were used. Differences in *D. viviparus* infection status of repeatedly sampled cows between both sampling periods (summer and autumn) were assessed using a McNemar’s test. Differences in larvae excretion between parities were analysed by using the two-tailed Fisher’s exact test. The relationship between BTM ODR levels and the percentage of cows showing larvae excretion in autumn was tested by using Spearman rank correlation. Pearson’s rank correlation was used to estimate the correlation between herd ODR value and herd size. The association between individual antibody levels in autumn (from cows with larvae excretion in summer) and FLC (full count data) in summer and autumn was tested by using Spearman’s rank correlation. Spearman’s rank correlation was also used to estimate the relationship between the percentage of coughing cows and the percentage of patent *D. vivparus*-infected cows in autumn. In addition, differences between coughing status (CS+ or CS-) within larvae excretion categories (FLC positive or negative) in autumn was tested by using a two-tailed Fisher’s exact test and the kappa index (index of coincidence). The kappa index takes values between -1 and +1, with small values indicating insufficient accordance between both observations. *P*-values ≤ 0.05 were considered significant for all analyses.

For test-day milk production parameters (dependent variables), a dataset was created including the first test-day after each parasitological examination of each cow. Records with a time span over 40 days between faecal sampling and test-day date were excluded from the analyses. Days in milk was classified into five lactation stage categories according to Huth [23]: ≤ 14, 14–77, 78–140, 141–231, and ≥ 232 days after calving. All cows as of parity five were classified in parity number > 4. In the multivariable analyses, FLC was binary coded as a potential influencing factor on milk production parameters. To assess the association between individual patent *D. viviparus* infection status (independent variable) and milk production parameters milk yield, milk protein and milk fat content (dependent variables) based on individual test-day records, linear mixed models (PROC MIXED) were applied. The effect of main interest was the *D. viviparus* infection status (larvae excretion or not). Other included effects were parity number (1, 2, 3, 4, > 4), lactation stage (≤ 14, 14–77, 78–140, 141–231, > 232 days after calving), genetic line (HF-NZ, HF-GHm, HF-GHp, DSN, Crosses), test-day season (records before September, records in September and later), and time span between each test-day record and faecal sampling date (≤ 8; > 8 and ≤16; > 16 and ≤ 24; > 24 and ≤ 32; > 32). Farm and cow were included as random effects in the model. Interaction terms between FLC and other fixed effects were estimated, but the analysis revealed no significances. Hence, no interaction term was included in the final mixed model analysis. The distribution of conditional residuals of all multilevel models was plotted to check the fit of the final models.

## Results

### Herd and individual faecal examinations

Of all herds examined for lungworm larvae, 18.8% (3/16) were positive in summer and 17.6% (3/17) in autumn 2015. The overall herd prevalence was 23.5% (4/17). Table 1 provides an overview of the coproscopical results of all farms. On one of the four positive farms, only one cow showed a patent *D. viviparus* infection in July and switched to a negative status in autumn. Cows with patent lungworm infections were primarily detected in farms with herd sizes of about 70 cows. The largest farm (Table 1, herd no. 3) frequently acquired additional cows from The Netherlands. On this farm, 2 of the 7 cows excreting larvae in summer had been recently acquired as well as 7 of 23 cows excreting larvae in autumn. The farm with the highest prevalence of up to 15.1% was a farm which did not introduce any new cows (Table 1, herd no. 6).

**Table 1:** Dairy cows excreting *D. viviparus* larvae (positive farms are marked in bold), BTM ELISA results (available for autumn only) and occurrence of coughing (our own and farmers’ observations; data available for autumn only)

Herd		Cows excreting lungworm larvae/total cows (%)		BTM ELISA ODR value	Occurrence of coughing and larvae excretion in autumn 2015			
No.	Size	Summer 2015	Autumn 2015		Farmer observation	Our observation		
					Occurrence of coughing	Occurrence of coughing	No. of coughing cows/total cows (%)	No. of coughing cows positive for larvae excretion
1	82	0/59 (0)	0/74 (0)	0.125	–	+	1/74 (1.4)	0
2	65	0/55 (0)	0/54 (0)	0.120	–	+	2/54 (3.7)	0
**3**	**215**	**7/164 (4.3)**	**23/195 (11.8)**	**0.342**	**+**	**+**	**25/195 (12.8)**	**3**
4	90	0/70 (0)	0/80 (0)	0.230	–	+	7/80 (8.8)	0
5	105	0/74 (0)	0/58 (0)	0.118	–	+	1/58 (1.7)	0
**6**	**70**	**1/68 (1.5)**	**8/53 (15.1)**	**0.236**	**–**	**+**	**1/53 (1.9)**	**0**
7	80	0/54 (0)	0/54 (0)	0.139	–	–	0/54 (0)	0
8	57	0/54 (0)	0/54 (0)	0.165	–	–	0/54 (0)	0
**9**	**70**	**nd**	**4/50 (8.0)**	**0.196**	**+**	**+**	**4/50 (8.0)**	**0**
10	16	0/15 (0)	0/15 (0)	0.113	–	–	0/15 (0)	0
**11**	**79**	**1/75 (1.3)**	**0/72 (0)**	**0.127**	**–**	**+**	**5/72 (6.9)**	**0**
12	71	0/60 (0)	0/67 (0)	0.132	–	+	6/67 (9.0)	0
13	32	0/26 (0)	0/31 (0)	0.146	–	–	0/31 (0)	0
14	31	0/30 (0)	0/27 (0)	0.216	+	–	0/27 (0)	0
15	48	0/44 (0)	0/44 (0)	0.148	–	+	2/44 (4.6)	0
16	77	0/76 (0)	0/68 (0)	0.148	–	+	6/68 (8.8)	0
17	40	0/36 (0)	0/32 (0)	0.152	+	+	1/32 (3.1)	0
Total	72.2^a^	9/960 (0.9)	35/1028 (3.4)	0.168^a^	4 CS+ / 13 CS-	12 CS+ / 5 CS-	61/1028 (5.9)	3/61 (4.9)

*Abbreviations*: *CS+* coughing positive, *CS-* coughing negative, *nd* not determined

^a^Mean, ^b^based on our observation; Farms with a positive coproscopy were marked in bold

**Fig. 1:**
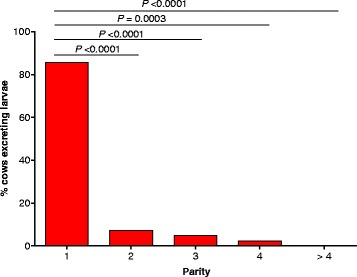
The distribution of cows with patent *D. viviparus* infection according to parity

In summer, 0.9% (9/960) of cows excreted *D. viviparus* larvae. Prevalences within parities were 2.4% (6/252) in parity 1, 0.0% (0/261) in parity 2 and 0.6% (1/158) in parity 3, while no infected cow was detected in parity 4 or > 4. In autumn, 3.4% (35/1028) of cows showed a patent *D. viviparus* infection and prevalences within parities were 11.1% (30/270) in parity 1, 1.1% (3/261) in parity 2, 0.5% (1/185) in parity 3, 0.8% (1/126) in parity 4 and 0.0% (0/185) in parity > 4. Most of the cows positive for larvae excretion in summer or autumn (*n* = 42) fell in parity 1. Respective percentages within different parity categories are presented in Fig. 1. In the repeatedly measured cows (*n* = 820), the prevalence was significantly higher in autumn with 3.2% (26/820) compared to summer 0.5% (4/820) (McNemar’s test = 15.75; *df* = 1; *P =* 7.229e-05). Regarding all individual cows (*n* = 1166), the overall prevalence was 3.6% (42/1166). Of these 42 patent infected cows, repeated measurements were feasible for 32 cows resulting in 6.3% (2/32) dairy cows with larvae excretion twice, in summer and in autumn. As shown in Table 2, FLC ranged between 0 and 46 in 40 g faeces. Faecal larvae counts > 10 were only found in cows which were in their first lactation.

**Table 2:** Number of patent infected dairy cows within different excretion categories in summer and autumn 2015

No. of lungworm larvae in 40 g of faeces	No. of cows in summer 2015 (*n* = 960)	No. of cows in autumn 2015 (*n* = 1028)
1–5	7	24
6–10	1	3
11–20	1	3
21–30	0	1
31–40	0	2
> 40	0	2
Total	9	35

### Herd management and occurrence of coughing

Four farmers stated that they observed the occurrence of coughing in the herd (Table 1). As shown in Table 1, in two of these herds patent lungworm infections were detected. No lungworm larvae were detected in the two other herds, although farmers observed coughing. According to own observations, 12 farms were assigned to a CS+ status, and all four farms on which larvae excretion was detected were considered as CS+. Three of the 17 farms noted that they frequently acquired additional cows from other regions in Germany. Moreover, one of these three farms acquired cows from the Netherlands. Patent lungworm-infected cows were detected in two of the three farms which had recently introduced additional cows. No significant association was observed between the percentage of coughing cows and the percentage of patently *D. viviparus*-infected cows on the 17 farms in autumn (Spearman’s rho = 0.33, *P* = 0.2039) (Additional file 1: Figure S1).

**Fig. 2:**
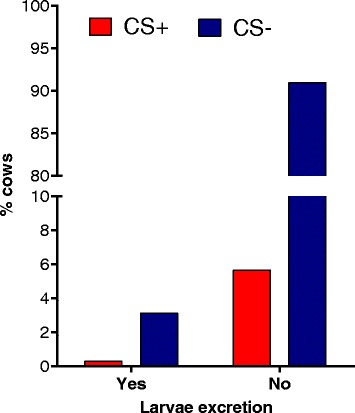
Percentage of coughing cows in autumn within larvae excretion categories. *Key*: CS, clinical signs; Yes, patent *D. viviparus* infection; No, no larvae excretion

**Fig. 3:**
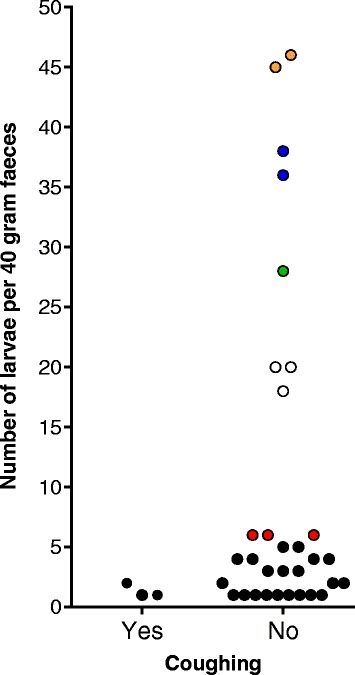
Number of larvae per 40 g of examined faeces within coughing categories in individual dairy cows in autumn. Different colours of points indicate larvae excretion categories according to Table 2. *Key*: Yes, coughing was observed in autumn; No, coughing was not observed in autumn

Coughing observation in autumn resulted in 5.9% (61/1028) of cows assigned to a CS+ status. When connecting coughing observation with results of parasitological examination in autumn, no significant difference in the proportion of CS+ and CS- assigned cows between patent infected and non-infected cows was observed (Fisher’s exact test, *P* = 0.4580) (Fig. 2). The level of agreement between patent *D. viviparus* infection status in autumn and coughing estimated by the Kappa method was 0.0220 (CL at 95%: -0.0485–0.0925). A concurrent presence of larvae excretion and coughing in the same cow was detected in only three animals. Thus, only 4.9% (3/61) of coughing cows showed a patent lungworm infection in autumn. Of these three cows, one cow excreted lungworm larvae in summer as well as in autumn. Figure 3 demonstrates that there was no association between coughing and the total number of larvae per 40 g in individual cows showing larvae excretion.

### BTM and individual milk samples

All BTM samples showed negative BTM ELISA results, values ranged between 0.113 and 0.342 ODR (cut-off value 0.410 ODR; [8]). The mean ODR value was 0.168. In autumn, a significant positive correlation between BTM ODR values and the percentage of cows excreting larvae in the herd was observed (Spearman’s rho = 0.61, *P* = 0.0074) (Additional file 2: Figure S2). Furthermore, a significant correlation between BTM ODR value and total herd size was found (Pearson’s *r* = 0.64, *P* = 0.0059).

From seven of the nine cows which were shedding larvae in summer, individual milk samples could be collected in autumn. For six of these cows FLC in summer was 1 larva per 40 g faeces, whereas one cow excreted 15 larvae/40 g faeces. In two of the six cows excreting 1 larva/40 g faeces in summer, larvae excretion was also observed in autumn (again 1 larva/40 g faeces). The ODR values of the individual milk ELISA of these six cows ranged between 0.114 and 0.288 ODR with a mean of 0.229 ODR (cut-off value 0.573 ODR; [17]). In the cow excreting 15 larvae/40 g faeces, a positive ELISA value of 0.744 ODR but no larvae excretion was observed in autumn. This cow was a recently acquired heifer on the largest farm (Table 1, herd no. 3).

### Milk production data

In total, 1532 test-day observations of 1003 cows were available and combined with parasitological examinations in the multivariable analysis. The average of milk yield was 21.4 (range: 2.4–47.6) kg/cow/day. The average milk protein content was 3.51% (range: 2.42–6.32%). The average milk fat content was 4.17% (range: 1.98–7.19%). Parity number ranged from 1 to 11 with a mean of 2.8. The average value for days in milk (DIM) was 190 days with a few cows showing very high DIM values (range: 1–741 days). The average lactation stage according to Huth [23] was 3.8 (range: 1–5). The average time span between test-day date and parasitological examination was 20.3 days (range: 0–40 days). Descriptive statistics for milk production parameters classified by *D. viviparus* infection status are shown in Table 3.

**Table 3:** Descriptive statistics for parity, days in milk (DIM), milk yield (kg/cow/day), milk protein content (%) and milk fat content (%) for *D. viviparus* positive and negative cows where test-day data was available [two records per cow if faecal examination and test-day data were available in summer and autumn (*n* = 1532); note that some cows changed infection status]

	*D. viviparus*-positive cows			*D. viviparus*-negative cows		
	Mean	SD	Range	Mean	SD	Range
Parity	1.3	0.7	1.0–4.0	2.9	1.8	1.0–11.0
DIM	116.1	55.4	19.0–254.0	191.8	119.5	1.0–741.0
Milk yield	19.9	5.5	9.4–34.4	21.4	7.1	2.4–47.6
Milk protein content	3.4	0.3	2.7–4.0	3.5	0.4	2.4–6.3
Milk fat content	4.1	0.5	3.3–5.6	4.2	0.7	2.0–7.2

*Abbreviation*: *SD* standard deviation

### Relationship between patent *D. viviparus* infections and milk production parameters

All results of the model output regarding the association between *D. viviparus* infection status and milk production parameters are presented in Table 4. Results for least-squares means and the test of significance for fixed effects (sum of squares type III) as included in the multivariable analyses are given in Additional file 3: Table S1. The average daily milk production was significantly lower (*P* = 0.0406) in cows with a patent *D. viviparus* infection compared to negative cows. Cows with a negative infection status yielded 23.4 kg milk/cow/day, while infected cows showed an average daily milk production of 21.8 kg milk/cow/day. All other included effects showed a significant influence on average daily milk production in the model analysis. No significant association was found between patent *D. viviparus* infections and milk protein content (*P* = 0.3666) or milk fat content (*P* = 0.6092).

**Table 4:** Estimates with corresponding confidence intervals (95% CI) and *P*-values for the milk production parameters milk yield (kg/cow/day), protein content (%) and fat content (%) from the multivariable linear mixed model

Production trait		Milk yield		Protein content		Fat content	
		Estimate (95% CI)	*P*-value	Estimate (95% CI)	*P*-value	Estimate (95% CI)	*P*-value
Intercept		16.69 (13.46; 19.93)	< 0.0001	4.06 (3.87; 4.24)	< 0.0001	4.87 (4.49; 5.25)	< 0.0001
FLC	FLC negative	1.62 (0.07; 3.18)	0.0406	-0.05 (-0.15; 0.05)	0.3666	-0.05 (-0.25; 0.15)	0.6092
	FLC positive	Baseline		Baseline		Baseline	
Parity	1	-3.49 (-4.38; -2.59)	< 0.0001	-0.00 (-0.06; 0.05)	0.9508	-0.02 (-0.13; 0.10)	0.7946
	2	-1.64 (-2.55; -0.72)	0.0005	0.09 (0.04; 0.15)	0.0017	0.03 (-0.09; 0.14)	0.6284
	3	0.80 (-0.15; 1.76)	0.1000	0.07 (0.01; 0.13)	0.0290	-0.06 (-0.18; 0.06)	0.2918
	4	0.71 (-0.33; 1.75)	0.1790	0.09 (0.02; 0.15)	0.0075	0.06 (-0.07; 0.19)	0.3784
	> 4	Baseline		Baseline		Baseline	
Lactation stage	DIM ≤ 14	10.20 (8.68; 11.71)	< 0.0001	-0.28 (-0.37; -0.18)	< 0.0001	-0.30 (-0.49; -0.10)	0.0026
	DIM 14-77	11.11 (10.39; 11.84)	< 0.0001	-0.62 (-0.67; -0.58)	< 0.0001	-0.63 (-0.72; -0.54)	< 0.0001
	DIM 78-140	8.35 (7.61; 9.08)	< 0.0001	-0.51 (-0.56; -0.47)	< 0.0001	-0.58 (-0.68; -0.49)	< 0.0001
	DIM 141-231	4.65 (4.03; 5.27)	< 0.0001	-0.25 (-0.29; -0.21)	< 0.0001	-0.31 (-0.39; -0.23)	< 0.0001
	DIM ≥ 232	Baseline		Baseline		Baseline	
Genetic line	DSN	-1.02 (-2.73; 0.69)	0.2406	-0.05 (-0.16; 0.05)	0.3008	-0.28 (-0.49; -0.08)	0.0067
	Crosses	0.33 (-1.93; 2.58)	0.7744	0.02 (-0.12; 0.16)	0.7717	-0.10 (-0.38; 0.19)	0.5039
	HF-GHm	1.45 (0.21; 2.68)	0.0223	-0.20 (-0.27; -0.12)	< 0.0001	-0.40 (-0.56; -0.25)	< 0.0001
	HF-GHp	0.94 (-0.64; 2.52)	0.2434	-0.15 (-0.24; -0.05)	0.0044	-0.33 (-0.52; -0.13)	0.0012
	HF-NZ	Baseline		Baseline		Baseline	
Test-day season	< September	1.39 (0.79; 1.98)		-0.23 (-0.27; -0.19)	< 0.0001	-0.26 (-0.34; -0.19)	< 0.0001
	≥ September	Baseline	< 0.0001	Baseline		Baseline	
Time span	≤ 8	-1.61 (-3.55; 0.31)	0.0998	-0.12 (-0.23; -0.01)	0.0581	-0.05 (-0.28; 0.18)	0.6782
	> 8 and ≤ 16	-3.71 (-5.54; -1.88)	< 0.0001	-0.09 (-0.20; 0.01)	0.1228	0.11 (-0.11; 0.33)	0.3169
	> 16 and ≤ 24	-2.72 (-4.47; -0.96)	0.0024	-0.04 (-0.14; 0.07)	0.5639	0.18 (-0.03; 0.39)	0.1014
	> 24 and ≤ 32	-2.49 (-4.67; -0.51)	0.0136	-0.05 (-0.17; 0.07)	0.5143	0.19 (-0.05; 0.43)	0.1180
	> 32	Baseline		Baseline		Baseline	
Random effects		Estimate		Estimate		Estimate	
Farm		7.97		0.01		0.06	
Cow		9.94		0.03		0.15	
Residual		13.41		0.06		0.22	

*Abbreviations*: *CI* confidence interval, *FLC* binary defined faecal larvae count (FLC = 0 classified as ‘FLC negative’; FLC ≥ 1 classified as ‘FLC positive’) as a parameter for *D. viviparus* infection status

## Discussion

Previous studies have shown a significant negative association between *D. viviparus* antibody levels and total milk production in dairy herds based on BTM ELISA results [14, 15]. However, data on the relationship between patent *D. viviparus* infections determined by faecal examinations and daily milk production in individual dairy cows are lacking. Hence, the presented field study was carried out to address this topic in individual Black and White dairy cows. Here, a significant relationship between patent *D. viviparus* infections and total milk production (as assessed on the first available test-day after parasitological examination) was observed in adult dairy cows (-1.62 kg/cow/day) during the months July to October. This result is in accordance with those obtained by Dank et al. [14], who estimated a loss of 1.01 kg/cow/day in summer and 1.68 kg/cow/day in autumn in dairy herds in The Netherlands by using BTM ELISA results. A decrease of 0.50 kg/cow/day in the annual average milk yield as a consequence of increased BTM ELISA ODRs in August was reported by Charlier et al. [15]. In another study, a reduced milk production of 4 kg/cow/day was observed after a lungworm outbreak on two Dutch dairy farms, which was confirmed by individual serum samples and the occurrence of clinical signs [5]. However, this value (4 kg/cow/day) may be overestimated since no other factors affecting milk production (e.g. lactation stage, parity, season of test-day sampling) were included in this calculation as conceded by the authors themselves [5]. Although most factors influencing milk production were taken into account in the present analysis, reduced milk yield might also be due to other (infective) diseases (e.g. mastitis, milk fever, ketosis) [24, 25], whose diagnosis was beyond the scope of this study.

No significant decrease in individual milk protein or milk fat content during a patent lungworm infection was found in the present analyses. Surprisingly, cows which excreted larvae showed slightly, but not significantly higher values for these production parameters. This finding is contrary to previous studies estimating the association between BTM ELISA results and milk quality parameters on herd level. A significantly lower milk fat content between 0.08–0.14% in BTM positive dairy herds compared to those with lower antibody levels, but no association between BTM status and milk protein content was observed by Dank et al. [14]. A drop of 0.02% in the annual milk fat and protein content as a consequence of increased BTM ELISA ODRs in August was detected by Charlier et al. [15]. Dank et al. [14] hypothesized that the reductions in milk fat content are the consequence of an energy-costly immune response against *D. viviparus* as shown in a previous experimental study in primary infected calves [26]. This was not confirmed in our study, which indicates that an energy-costly immune response might be only occurring in primary infected heifers or calves. A possible explanation for the not significant, but trend to higher milk protein and fat content in patent *D. viviparus*-infected cows in the presented study lies in the naturally negative correlation between milk yield with protein and fat content, as a decline in milk yield (as estimated in patent *D. viviparus*-infected cows in this study) is accompanied by a higher milk protein content and a higher milk fat content [27, 28]. It remains unknown, whether or when milk yield in patent infected cows regain those of negative cows. Hence, it would be interesting to estimate the association between milk production parameters and monthly results of faecal examinations, which may be supported by serological assays, on an individual dairy cow level.

Overall, detecting the impact of patent *D. viviparus* infections on milk production parameters is hampered by the fact that both commonly used methods (serology and faecal examination) for the assessment of dictyocaulosis are linked to some disadvantages. Faecal examinations are not as sensitive as serological assays [10, 17, 29], but represent a better indicator of an actually existing lungworm infection compared to *D. viviparus* antibody titres, which persist up to 7 months after infection [16, 17]. Therefore, the association between milk production and milk quality was estimated by cows positive in faecal examination. However, the results of the presented study are limited by the fact that some cows were possibly detected as false-negative, since they might have been in the prepatent period or due to the low sensitivity of the Baermann technique. The latter disadvantage we aimed to overcome by examining a large amount of faeces (40 g per cow). Nevertheless, in most cows FLC was only 1–5 larvae per 40 g faeces, which is in accordance to other authors [8, 9]. Hence, patent *D. viviparus* infections are often missed by faecal examinations, particularly when using small amounts of faeces. Another reason for the low prevalence in our study, which is comparable to those reported by Schunn et al. [8], are reinfection events, which can be assumed for at least some of the herds included in the study. In animals, which have acquired immunity due to previous infections or vaccination, lungworm reinfections result in low or lacking larvae excretion [19, 30, 31]. Continuous reinfection over years might strengthen the protective immunity as larvae excretion was only observed in parities 1 to 4, although cows between parity 1 and 11 were included in the presented study. This outcome corroborates with findings of Ploeger et al. [10], who found most coproscopical positive cows in parity 1. In our study, the largest number of cows excreting larvae was found in one of the largest farms. This farmer purchased heifers, which had no previous access to pasture in summer preceding the examinations presented here. The heifers’ first pasture access was approximately 1 month after purchase.

Herd antibody levels measured by BTM ELISA are helpful to evaluate if an infection with *D. viviparus* was present within the last months, but they do not necessarily indicate a concurrent lungworm infection. Additionally, the MSP used as diagnostic ELISA antigen is only expressed by adult male worms. Thus, detection of antibodies with the BTM ELISA is dependent on patent infections with antibody levels reaching the cut-off as of days 26–50 post-infection [17, 19, 32]. Larvae excretion in the absence of seropositivity was observed in individual cows and explained by just recently ingested larvae or by a low sensitivity of the ELISA under field conditions [10]. Recently, antibody levels in different (re)infection scenarios were evaluated by Strube et al. [19]. While the infection dose had no influence on antibody development, antibody levels were reduced in magnitude and duration or even lacking following *D. viviparus* reinfection of (partially) immune animals, while low levels of larvae excretion can occur [19]. The suspected low BTM ELISA sensitivity in the field is thus attributable to the shortened seropositivity period of previously infected animals. Indeed, longitudinal field studies on lungworm-positive dairy farms showed that the majority of individual as well as BTM ELISA samples were positive for one or two consecutive months only [8, 19]. These findings corroborate with the results of the presented field study, where a positive individual milk ELISA result (≥ 0.573 ODR; [8, 17]) in autumn was detected in only one of the seven cows which were excreting larvae in summer. This ELISA-positive animal was recently acquired by the farmer. Additionally, it showed the highest level of larvae excretion in summer (8 weeks before serological testing), but no larvae excretion in autumn. No reliable statement can be made whether this cow was primary infected after introduction into the herd, since ELISA was exclusively carried out in September. Regarding BTM ELISA, no farm was tested positive. These findings are in accordance with findings by Schunn et al. [8], who stated that a within-herd-prevalence of 20% is required to reliably exceed the cut-off value of 0.410 ODR in the BTM ELISA.

Based on our dataset, a significant positive correlation (0.64) was detected between herd size and BTM ODR value, whereas Dank et al. [14] reported that BTM negative herds were significantly larger. In another study, antibody levels were higher in large herds and those which frequently purchased new animals [15], which was explained by a larger number of transmission possibilities. Since the presented study was designed on an individual cow level with a small BTM sample size (*n* = 17), our results are not directly comparable to herd-level studies including a larger BTM sample size.

Lungworm infections in cattle are often suspected due to the clinical symptom “coughing”. Hence, some studies used coughing as a gold standard, e.g. to determine the sensitivity and specificity of the BTM ELISA [20]. However, based on the assumption that many dairy herds experience continuous reinfections over years and thus have developed immunity protecting from clinical disease, it remains equivocal if coughing is a reliable parameter for diagnosing lungworm infections. Thus, the present study aimed for the first time to assess the congruence of patent *D. viviparus* infections and coughing as a possible clinical symptom, including the observations of farmers and our own observations. Ploeger et al. [10] reported that farmers’ observation for the occurrence of coughing was in accordance with the authors’ assessment. This was not the case in the present study: more than half of all herds were regarded as CS+ in own assessments, while the farmers did not observe any clinical signs in their herd. In two farms, both coughing and larvae excretion was observed, while the farmer reported no conspicuous clinical symptoms. Hence, the use of farmers’ observations to detect coughing in a herd should be treated with caution. Otherwise, the threshold of farmers may be conceivably higher to classify mild coughing in the herd as a noticeable clinical symptom. Additionally, in our own assessment particular attention was drawn to coughing, whereas the farmer was not advised to focus on coughing but answered our question retrospectively.

Regarding the congruence of coughing and proven lungworm infection, only three of the 61 coughing cows showed larvae excretion simultaneously (4.9%). Results of the Kappa method were close to zero, indicating low coincidence. In addition, no significant difference between the proportion of cows excreting larvae and the proportion of coughing cows was found. Hence, coughing is not a reliable indicator of lungworm infections. Indeed, similar symptoms in dairy cows may also be caused by other respiratory tract infections, e.g. viral [33, 34] or bacterial infections, in particular with *Pasteurella* spp. ([5]; personal communication with Martina Hoedemaker, Clinic for Cattle, University of Veterinary Medicine Hannover, Germany, and Berit Kemper, Veterinary Praxis Dr. Berit Kemper and Holger Seth, Hemmoor, Germany). Such infective but also possible non-infective agents need to be considered as differential diagnosis in clinically lungworm-suspected cows.

## Conclusions

In conclusion, a significant negative association between *D. viviparus* infection status measured by faecal larvae counts and average daily milk production was observed. The majority of cows in this study appeared to be reinfected as herd and individual antibody levels did not reach the cut-off in most cases. Hence, during reinfections in adult dairy cows, immune responses seem to be sufficient to prevent effects on milk protein and fat content, but may not be adequate to keep total daily milk production at a constant level. Additionally, lower milk production due to a patent *D. viviparus* infection was not necessarily accompanied by the presence of clinical symptoms such as coughing. Only three animals showed both larvae excretion and coughing, reflecting that coughing is an unreliable indicator to detect lungworm infections in non-naïve dairy herds. Farmers and veterinarians should bear in mind that in dairy cows presenting coughing other differential diagnoses need to be considered in addition to dictyocaulosis.

## Additional files

Additional file 1: Figure S1. Correlation between the percentage of coughing cows and the percentage of patent *D. viviparus-*infected cows on the 17 farms in autumn 2015. (PDF 23 kb)

Additional file 2: Figure S2. Correlation between the percentage of cows excreting larvae and BTM ELISA ODR from the 17 farms in autumn 2015. (PDF 27 kb)

Additional file 3: Table S1. Least-squares means with corresponding standard error (± SE) and *P*-values (Type 3 test of fixed effects) for the production traits milk yield, milk protein content and milk fat content within fixed effect classes. (DOCX 19 kb)

## Abbreviations

BTM: Bulk tank milk
CS: Coughing status
DIM: Days in milk
ELISA: Enzyme-linked immunosorbent assay
FLC: Faecal larvae count
GHC: German Holstein cow
MSP: Major sperm protein
ODR: Optical density ratio
