# Supplementary material for: The effect of patent Dictyocaulus viviparus (re)infections on individual milk yield and milk quality in pastured dairy cows and correlation with clinical signs
Source: Parasit Vectors. 2018 Jan 8;11:24. doi: 10.1186/s13071-017-2602-x (PMC5759297; doi:10.1186/s13071-017-2602-x)
Supplement: Supplementary file 2 — Correlation between the percentage of cows excreting larvae and BTM ELISA ODR from the 17 farms in autumn 2015. (PDF 27 kb) [file 13071_2017_2602_MOESM2_ESM.pdf]

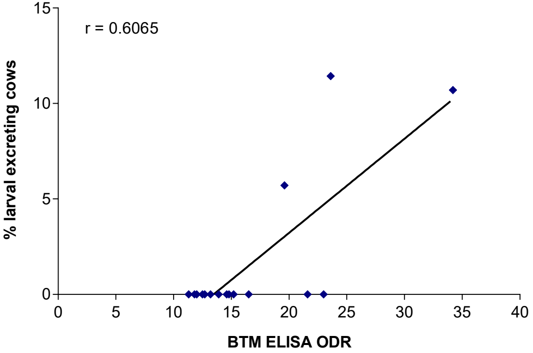

Additional file 2: Correlation between the percentage of cows excreting larvae and BTM ELISA ODR from the 17 farms in autumn 2015.
