# Supplementary material for: The effect of patent Dictyocaulus viviparus (re)infections on individual milk yield and milk quality in pastured dairy cows and correlation with clinical signs
Source: Parasit Vectors. 2018 Jan 8;11:24. doi: 10.1186/s13071-017-2602-x (PMC5759297; doi:10.1186/s13071-017-2602-x)
Supplement: Supplementary file 3 — Least-squares means with corresponding standard error (± SE) and P-values (Type 3 test of fixed effects) for the production traits milk yield, milk protein content and milk fat content within fixed effect classes. (DOCX 19 kb) [file 13071_2017_2602_MOESM3_ESM.docx]

Additional file 3: Table S1. Least-squares means with corresponding standard error (± SE) and *P*-values (Type 3 test of fixed effects) for the production traits milk yield, milk protein content and milk fat content within fixed effect classes.

| Fixed effects |  | Milk yield | | Protein content | | Fat content | |
| --- | --- | --- | --- | --- | --- | --- | --- |
|  |  | Least-squares means (± SE) | *P*-value | Least-squares means (± SE) | *P*-value | Least-squares means (± SE) | *P*-value |
| FLC | FLC negative | 23.38 ± 0.76 | 0.0406 | 3.47 ± 0.03 | 0.3666 | 4.18 ± 0.07 | 0.6092 |
|  | FLC positive | 21.76 ± 1.09 |  | 3.52 ± 0.06 |  | 4.23 ± 0.12 |  |
| Parity | 1 | 19.81 ± 0.86 | ≤ 0.0001 | 3.45 ± 0.04 | 0.0002 | 4.19 ± 0.09 | 0.3157 |
|  | 2 | 21.66 ± 0.88 |  | 3.54 ± 0.04 |  | 4.24 ± 0.09 |  |
|  | 3 | 24.10 ± 0.90 |  | 3.51 ± 0.05 |  | 4.14 ± 0.09 |  |
|  | 4 | 24.00 ± 0.94 |  | 3.54 ± 0.05 |  | 4.27 ± 0.10 |  |
|  | >4 | 23.29 ± 0.92 |  | 3.45 ± 0.05 |  | 4.21 ± 0.10 |  |
| Lactation stage | DIM ≤14 | 25.91 ± 1.12 | ≤ 0.0001 | 3.55 ± 0.06 | ≤ 0.0001 | 4.28 ± 0.13 | 0.0001 |
|  | DIM 14-77 | 26.82 ± 0.87 |  | 3.21 ± 0.04 |  | 3.94 ± 0.09 |  |
|  | DIM 78-140 | 24.06 ± 0.86 |  | 3.32 ± 0.04 |  | 3.99 ± 0.09 |  |
|  | DIM 141-231 | 20.36 ± 0.86 |  | 3.58 ± 0.04 |  | 4.26 ± 0.09 |  |
|  | DIM >232 | 15.71 ± 0.87 |  | 3.83 ± 0.04 |  | 4.57 ± 0.09 |  |
| Genetic line | HF-NZ | 22.23 ± 1.00 | 0.0010 | 3.57 ± 0.05 | ≤ 0.0001 | 4.43 ± 0.11 | ≤0.0001 |
|  | HF-GHm | 23.68 ± 0.84 |  | 3.38 ± 0.04 |  | 4.03 ± 0.09 |  |
|  | HF-GHp | 23.17 ± 1.02 |  | 3.43 ± 0.05 |  | 4.12 ± 0.11 |  |
|  | DSN | 21.21 ± 0.95 |  | 3.52 ± 0.05 |  | 4.15 ± 0.15 |  |
|  | Crosses | 22.56 ± 1.27 |  | 3.59 ± 0.07 |  | 4.34 ± 0.15 |  |
| Test-day season | <September | 23.27 ± 0.87 | ≤ 0.0001 | 3.38 ± 0.04 | ≤ 0.0001 | 4.07 ± 0.09 | ≤ 0.0001 |
|  | ≥September | 21.88 ± 0.88 |  | 3.61 ± 0.04 |  | 4.34 ± 0.09 |  |
| Time span | ≤8 | 23.06 ± 0.92 | ≤ 0.0001 | 3.44 ± 0.04 | 0.0385 | 4.08 ± 0.09 | 0.0003 |
|  | > 8 and ≤16 | 20.97 ± 0.88 |  | 3.46 ± 0.05 |  | 4.24 ± 0.09 |  |
|  | >16 and ≤24 | 21.96 ± 0.89 |  | 3.52 ± 0.05 |  | 4.30 ± 0.09 |  |
|  | >24 and ≤32 | 22.19 ± 0.97 |  | 3.51 ± 0.04 |  | 4.32 ± 0.11 |  |
|  | >32 | 24.68 ± 1.15 |  | 3.56 ± 0.06 |  | 4.13 ± 0.13 |  |
